# Growing Up in a Digital World **–** Digital Media and the Association With the Child’s Language Development at Two Years of Age

**DOI:** 10.3389/fpsyg.2021.569920

**Published:** 2021-03-18

**Authors:** Annette Sundqvist, Felix-Sebastian Koch, Ulrika Birberg Thornberg, Rachel Barr, Mikael Heimann

**Affiliations:** ^1^Infant and Child Lab, Department of Behavioural Sciences and Learning, Linköping University, Linköping, Sweden; ^2^Department of Psychology, Georgetown University, Washington, DC, United States

**Keywords:** digital media, joint media engagement, technoference, Language ENvironment Analysis, language development, parent-child turn-taking

## Abstract

Digital media (DM), such as cellphones and tablets, are a common part of our daily lives and their usage has changed the communication structure within families. Thus, there is a risk that the use of DM might result in fewer opportunities for interactions between children and their parents leading to fewer language learning moments for young children. The current study examined the associations between children’s language development and early DM exposure.

**Participants:** Ninety-two parents of 25months olds (50 boys/42 girls) recorded their home sound environment during a typical day [Language ENvironment Analysis (LENA)] and participated in an online questionnaire consisting of questions pertaining to daily DM use and media mediation strategies, as well as a Swedish online version of the MacArthur Communicative Development Inventory, which includes a vocabulary scale as well as a grammar and pragmatics scale.

**Results:** Through correlations and stepwise regressions three aspects of language were analyzed. The child’s vocabulary was positively associated with interactional turn-taking. The child’s vocabulary and grammar were negatively associated with the likelihood of parent’s device use during everyday child routines and the amount of TV watched by the child. The child’s pragmatic development was also positively associated with the parent’s device use in child routines but also with the parent’s joint media engagement (JME), as well as the child’s gender (where girls perform better).

**Conclusion:** Our study confirms that specific aspects of the 2-year old’s DM environment are associated with the child’s language development. More TV content, whether it is viewed on a big screen or tablet, is negatively associated with language development. The likelihood of parents’ use of DM during everyday child routines is also negatively associated with the child’s language development. Positive linguistic parental strategies such as interactional turn-taking with the child, JME, and book reading, on the other hand, are positively associated with the child’s language development.

## Introduction

Living in a digital world is changing the way we interact with each other, for children and adults alike. This may consequently change the way young children, growing up today, acquire language, as language development is dependent on the linguistic input achieved through the interactions that occur during child-adult conversations (e.g., Tomasello, 2003; Romeo et al., 2018). The limited empirical research has, thus far, investigated associations between television exposure and conversational turns in language input and shown a negative association (Christakis et al., 2009). Despite rapid changes in the availability of digital media (DM) in the home, including Smartphones, television, gaming consoles, and tablets, as well as a number of digital services like streaming television and social media, little research has examined whether DM in the home is associated with child-adult conversations. The child-adult conversations may, in turn, be associated with child language, and foremost vocabulary. The present study focuses on the importance of child-adult interactions to language development and how this development may be associated with the use of DM and digital devices in the home environment.

### Development of Language

Early social interactions between the adult and child shape and act as the foundation of the child’s linguistic learning (Golinkoff et al., 2019). Previous studies have shown that parent-child interactional turn-taking is of importance for the child’s language development, more so than the number of words the child hears (Romeo et al., 2018). Making time to engage with the child, conversing back and forth, describing and explaining the child’s activities, actions and thoughts will expand and develop the child’s linguistic capacity and understanding (Meins and Fernyhough, 1999; Tamis-LeMonda et al., 2018; Golinkoff et al., 2019).

When parents converse with their young child, they will often use child-directed language (Westerlund and Lagerberg, 2008). Studies of the parent’s child-directed speech suggest several important aspects. The key ingredients are that the language responses the child encounters must be immediate, reliable, and accurate in content and relevance (Roseberry et al., 2014), which assumes that the parent is attentive and not preoccupied. This attentive communication style maintains an environment that is stimulating for language development (Karrass et al., 2003) and can occur during frequent routines such as book reading, car trips, feeding, and potty-training (Bruner, 1983; Zimmerman et al., 2009). Book reading for small children generally occurs in a dialog with the child; maintaining child-directed speech, stopping to explain matters the adult decides to explain, or to expand on matters the child questions. Positive effects from picture book reading or e-book reading are often attained through the interactions between the child and the adult (Kucirkova, 2019). It is the interactive aspect of reading that is of utmost importance for linguistic development (Mol et al., 2008). Unlike a TV show or a YouTube clip, an adult who reads to a child is enabling the child to listen at his or her own pace and perhaps rehearse and repeat words and actions depicted in the book and for adults to broaden the understanding of the topic by focusing on the child’s current understanding. When parents pause a recorded TV episode similar effects are observed (Strouse et al., 2013).

A common way to describe language is through the three concepts of form (e.g., syntax), content (e.g., vocabulary), and use (pragmatics; Bloom and Lahey, 1978). Often described through a Venn diagram where each concept has its unique characteristics, but they also share characteristics. Thus, each is a synergetic system, each concept developing independently, but acting as an integrative whole (ASHA, 1993). Consequently, when analyzing the development of language, one should take into account that different aspects in the child’s development or different aspects in the environment may differentially affect the form, content, or use of language (Bloom and Lahey, 1978).

### Digital Media and Family Life

Digital media fulfill diverse functions in parents’ and families’ lives and DM are used in as many as 98% of families with children in Sweden [Swedish Media Council (SMC), 2019]. This may lead to an overuse of DM, and users describe an attraction to DM including a desire to repeatedly check their social media or mail for possible updates, a desire they find hard to resist even in situations when the parents are spending time with their child (Oulasvirta et al., 2012). Adult DM use may hinder interactions between the child and the adult, thus affecting the language development of the child. If the child also independently uses DM, the child’s DM use is also a factor that changes the child’s language learning environment.

Disruption in interactions due to DM by parents or by children has been termed *technoference* (Reed et al., 2017, McDaniel and Radesky, 2018a,b, Sundqvist et al., 2020). This may occur in any situation where the adult and the child are interacting, such as during play-situations, book reading, or meal-time (Radesky et al., 2014; McDaniel and Radesky, 2018a,b). Parents estimated that an average of four instances of technoference occurred per day, where the parent’s DM use accounted for the majority of instances of technoference (Sundqvist et al., 2020). The exact number may be much higher as this can be hard to remember retrospectively (see Barr et al., 2020). Christakis et al. (2009) showed that the parents’ interactions with their child decreased as the digital sounds, predominantly television sounds in the vicinity of the child, increased. Additionally, a recent meta-analysis has shown that a greater quantity of screen use during infancy may be related to a delay in language development (Madigan et al., 2020).

The amount of DM and the occurring incidences of technoference is of importance, but research has shown that it is equally important to analyze if and how the parents use media together with the child (Nathanson, 2015). Research suggests, specifically, that parental mediation of children’s media use, such as discussing the content with the child, may reduce negative associations with DM usage (Nathanson, 2015; Madigan et al., 2020). This type of interaction is termed *joint media engagement* (JME) and shapes how children will respond to and use media. Higher JME has been associated with positive outcomes in family connectedness (Padilla-Walker et al., 2012) increased infant attention and responsiveness (Barr et al., 2008) and increased infant learning from touchscreens (Zack and Barr, 2016). Valkenburg et al. (1999) describe three different parental mediation styles restrictive mediation, instructive mediation, and social co-viewing. Instructive mediation describes parental behaviors that are comprised of JME including discussions with the child during and about the media activities. Social co-viewing represents behaviors in which the parent co-view media together but without the joint engagement in the DM task. Finally, restrictive mediation entails the parent enforcing device/content rules and/or time allowances connected to DM use.

Various family routines and activities which promote interaction between the child and the adult such as increased conversations in ordinary situations with the child, book reading (paper or e-books), and JME when using DM may be conducive for the child’s language development (Nathanson, 2015). It has been suggested that technoference or excessive child solitary use of DM decreases or disrupts typical interactions between adults and children, essential for language development, and consequently interferes with language development (Zimmerman et al., 2009).

Digital media exposure often reduces child-adult interaction because DM does not facilitate socially contingent conversational turns with a language partner whose responses are immediate, reliable, and accurate in content and relevance (Anderson and Hanson, 2017). Although some parents provide descriptions and even pause the video to discuss content with some success, such language-rich interactions tend to occur less often than during face-to-face interactions (Strouse et al., 2013). Without the support of an actively and instructively mediating adult, DM alone cannot repeat or explain the content according to the child’s specific needs. It is, thus, possible that solitary DM engagement, by the parent or by the child, will be negatively associated with the child’s language development. The use of DM may consequently limit the interactions between the child and the adult. It is, nevertheless, possible that even though the child or the adult uses DM, they could still maintain a high level of adult-child interaction, which would be associated with positive language outcomes. Examples of activities that support an increased interaction and thus would be positively associated with child language development are for instance JME and book reading (paper/e-books). Most studies of young children, so far, have examined how DM is associated with one specific aspect of language (for instance vocabulary) or a more general language measure (Madigan et al., 2020). We hypothesized that different aspects of language may be differentially associated with types of DM usage and this study will examine three aspects of language (content, form, and use of language) in 2-year olds who are at the early stages of expressively using grammar, vocabulary, and pragmatics. It is important to note that DM in the context of this study will include all digital devices that enable the display of the audio-visual output: TV, smartphones, tablets, and computers. Using today’s technology, casting your favorite music to a TV screen or watching TV on a smartphone is just as common as watching broadcast TV on a TV. Different devices do not necessarily display different content.

Our study asks three questions: (1) What are the characteristics of the child’s and parent’s DM use and what are the characteristics of children’s language development and home sound environment? We hypothesized that 2-year-olds would use DM on a daily basis and that parents would use their own device during child routines. We also hypothesized, given the nature of our sample, that their language development would be in the typical range for Swedish children. (2) Are 2-year-old’s DM use and parents’ DM use associated with the child’s language development? Based on the research on child DM use, we hypothesized that an increase in the family DM use would be negatively associated with child language development, specifically vocabulary. Based on the research on technoference, we hypothesized that a higher likelihood of parental DM use during ordinary child routines would be negatively associated with the child’s language development. Based on research on background TV use, we hypothesized that background TV would be negatively associated with the child’s language development. We did not have specific hypotheses for whether each of these variables would be associated with form, content, and use, except for predicting that higher child DM use would be associated with poorer vocabulary. (3) Are factors in the home sound environment such as increased interaction (adult-child turn-taking), increased book reading (e-books or paper books), and JME (interactional mediation) positively associated with the child’s language development? We hypothesized that increased interaction, book reading, and JME, respectively, would be positively associated with the child’s language development.

## Materials and Methods

### Participants

Parents were invited by mail to participate with their child in a study at the Baby and Child Lab, Linköping University. All children (*N* = 1,324) born within a specified time period within the Linköping municipal area were invited by letter. Addresses were obtained from Statens personadressregister, a database that includes everybody that is registered as resident in Sweden. Those who expressed an interest were contacted by phone and informed about the current study. This study which commenced when the children were 9months of age (*N* = 127) is part of a larger study which also entails laboratory testing of the child. In the second wave of this study, at 2years of age, 92 families participated. The families that took part in this study all spoke Swedish with their child. In three homes another language was also spoken. Based on a well-baby clinic visit, all children were reported to be typically developing. This article presents data collected from 2018 to 2019 when children were 2years old. The families that took part in the current study all spoke Swedish with their child. In three homes another language was also spoken. Based on a well-baby clinic visit, all children were reported to be typically developing. This article presents data collected from 2018 to 2019 when children were 2years old.

Parents: The questionnaires were filled out by the 92 parents (80% mothers/20% fathers) at home, some parents did not answer certain questions, leading to missing data on some questions. The sample is noted in relation to specific questions. They were well educated (12% high school, 6% completed vocational training, 73% had completed university, and 9% held a Ph.D.).

Children: The 92 children (50 boys/42 girls) were on average 25.1months old (*SD* 0.31months). At the time of the study, 51% of the children did not have any siblings and 42% had one sibling.

In Sweden, there is a state-subsidized childcare program in which 100% of the children in this sample attended. The Swedish parental-leave system allows the parents to stay at home with their child for over a year from birth with 80% of their wages. When the child starts childcare the parents also have paid sick days if the child is sick, as well as on average 25days of paid vacation/year. All the children in this study attended childcare, and 75% attended more than 20h a week, which is common in this age group in Sweden (Swedish National Agency for Education, 2013). Even though most children spend several hours a week in childcare, the time they spend in the home is a significantly greater proportion of the time.

Digital media was available in all households and used by the parents as well as actively used by the children, at least once within the last 2weeks. Ninety-five percent of the households had a TV (used by 87% of the children), all of the households had smartphones (used by 100% of the children), 95% of the households had a computer (used by 93% of the children), 81% of the households had a tablet (used by 81% of the children), and 30% of the households had a DVD-player (used by 28% of the children). The frequency of digital device use in this sample is in line with other reports of digital device usage in Sweden (SMC, 2019).

### Procedure

After the phone call with the parents when information regarding the study was communicated, an email with a link to the questionnaire was mailed out to the parent as well as a letter containing instructions and the home recording device Language ENvironment Analysis, LENA (LENA Foundation, 2020).

Parents were instructed to choose a typical day, when the child was not attending childcare, and they would be spending time at home with their child for the recording of their child’s home sound environment (21% chose a weekday and 79% of the parents chose a weekend/holiday for the recording). The reasons for choosing a day when the child was not attending childcare were twofold, first, we would receive a recording of a typical day when the child spends time with their family and second, it was not feasible to receive consent from all the different preschools and all the different parents to record during a preschool day. The curriculum of all the childcare facilities is governed by the national school law and curricula were, therefore, likely to be similar to each other. The home sound environment at 2years of age is also likely to be similar to the first 17months of the child’s life when most of the children had not yet started childcare. When the child woke up in the morning, the parent was instructed to place the LENA recording device in the designated pocket on a specifically provided vest and to push the record button and leave it recording the whole day. The recorder turned off automatically after 16h. When asked which parent takes the most responsibility for the child during a day off from preschool, 40% reported that the mother takes most of the responsibility, 26% reported that the father takes most responsibly, and 29% reported that both parents take an equal amount of responsibly for the child.

The questionnaires were administered through Qualtrics™ software (Qualtrics, Provo, UT, United States) which is a research software company offering online data collection. The media questions in the questionnaire were developed by the Comprehensive Assessment of Family Exposure (CAFE) Consortium. The original media questions were in English and were translated to Swedish and back-translated by a native English speaker. The questionnaires were also pilot tested to make sure the questions were phrased and understood properly by parents with children in a similar age group. The CAFE consortium is an interdisciplinary international network of researchers that has developed a reliable and valid tool for assessing DM during childhood (Barr et al., 2020). The focus for the present study is the children’s language development assessed through the Swedish version of MacArthur Communicative Development Inventory, the Swedish Early Communicative Development Inventory – Words and Sentences (SECDI-2), and assessment of the parents and children’s media use and strategies through the CAFE questionnaire. Although the questionnaire was self-administered online, a lab visit occurred shortly after the administration of the online questionnaires and parents ask questions as needed regarding the questionnaire during the lab visit.

### Ethics

Written informed consent was obtained from the caregivers. The regional ethical review board, Linköping, Sweden, approved this study (2016/490-31 and 2018/609-32).

### Online Questionnaire

To address our research question, the following media questions from the CAFE battery (version 2) were analyzed:

#### Estimated Daily Use of Media

A daily estimated measurement of how much time the parent and the child spent using media. We asked how much time during a regular weekday the parent would spend doing one of six different activities, watching TV/DVD, using computers, reading books, using a tablet, using a smartphone, and playing video games on a play console. Parents choose from the following options: not at all, <30min, 30–60min, 1–2h, or >2h. The parents were asked to answer the same questions but with regards to their child as well.

Additionally, we asked: Has your child ever used mobile digital devices (smartphone, tablet, etc.) to do any of the following activities? View TV shows or movies, play games, use apps that are not games (e.g., FaceTime), listen to books, listen to music on digital devices. The choices were (1) we do not have the device, (2) never or less than once a week, (3) once a week, (4) 2–4 times a week, (5) daily, and (6) several times a day.

#### Digital Media Use During Child Routines

The likelihood that parent’s used digital devices during common everyday routines when their child was present were also estimated using a Likert type scale (I never do this, not very likely, neutral, likely, very likely, represented by values 1–5, respectively). The questions asked were: There are often times when parents have to use their smartphone or tablet when spending time with their child. How likely are you to use your phone or other devices (e.g., to make calls, text, check email, watch a video): (1) During meals, (2) getting your child ready for childcare, (3) during playtime, (4) during the bedtime routine, (5) while driving them to or from activities or when riding on public transportation. For the analysis, the mean value of the five routine activities, representing the most common DM behavior pattern of the parent was chosen, with higher values indicating higher DM usage during their children’s activities. The parents were also asked about if they had the TV on in the background while no one was watching (with the option never, almost never, sometimes, often, or always).

#### Media Mediation Strategies (an Adapted Version of the Valkenburg Scale)

This scale is aimed at analyzing how parents mediate their children’s media access and use (Valkenburg et al., 1999). The scale was translated from English to Swedish and adapted for today’s media environment. Originally, the scale focused on TV watching, but the scope of the questions was broadened in the current study to include other types of media, such as smartphones and tablets. The three different styles of parental mediation in media viewing that the scale covers are (a) Restrictive mediation where the parent prohibits viewing according to a set of rules, (b) Instructive mediation which henceforth will be called JME, where parents explain and discuss aspects of the media, and (c) Social co-viewing where parents and children simply watch together. Each of the styles is assessed and scored through five separate questions and the parents grade the likelihood of the mediation style on a Likert scale from never to always (1–5) from which a mean value for each strategy is calculated.

### Language Development SECDI-2

A Swedish online version of the MacArthur Communicative Development Inventory, the SECDI-2, was used to assess productive vocabulary, grammatical use, and pragmatic use. Good validity and reliability of the Swedish SECDI-2 have been established for the 16- to 28-month age group and the test-retest reliability of SECDI-2 is close to or above 0.90 (Berglund and Eriksson, 2000). SEDCI-2 contains: (A) Vocabulary production checklist (710 frequent Swedish words), (B) Feedback morphemes (words that signal that one has understood the interlocutor like yeah, mmh, or no), (C) Pragmatic scale which includes questions about how the child uses language. For instance, one question is “Does your child point to someone’s object and say the name of that person? For instance, the child could point to daddy’s bike and say: ‘daddy’” (max 10 points), (D) Grammar scale estimates how the child uses, for instance, plural, past tense, possessive inflections (max 12 points), *E. Maximum* length utterance of the child. We have chosen three subscales for analysis tapping Content (Vocabulary), Form (Grammar), and Use (Pragmatics) The subscales B and E were not included in the present analysis.

### Home Sound Environment

The naturalistic home language environment was recorded during a whole day using LENA. The recorder is a small device that fits into a pocket of a specially designed vest the child uses and records up to 16h of the child’s sound surroundings (LENA Foundation, 2020). The audio recordings are downloaded and analyzed through LENA Software Advanced Data Extractor (ADEX). The ADEX automatically categorizes the data according to preset algorithms into a variety of different variables; e.g., child speaker, female speaker, male speaker, duration of speech, and distant speech (LENA Foundation, 2020). The Lena’s segmentation and labeling process are designed to identify the dominant sound source in the child’s environment. This is accomplished in intervals of 800 milliseconds. In a segment, for instance, where the child is playing with his parent with the TV in the background for 5min. LENA will add all the intervals where the TV is dominant, where the key child’s voice is dominant, and where the adult voice is dominant. Algorithms also automatically calculate conversational turns, where there were <5s of silence or other sounds (i.e., in this example the TV) between the child’s and the adult’s utterances (LENA Foundation, 2020).

The variables included in the current analysis are: (1) Adult word count which is the number of adult words spoken (female and male voices) to and near the child; (2) Target child’s vocalizations/words; (3) Interactional turn-taking is the total number of conversational interactions between the child and an adult where one speaker initiates and the other responds within 5s. All recordings that were longer than 10h were selected for analysis (LENA Foundation, 2020). Due to technical difficulties, one recording was only 4h and was not included in the final sample. The reliability of LENA was originally established for American English but has also been evaluated in other languages, such as Swedish (Schwarz et al., 2017), showing that comparison within the same language will yield comparable results.

### Statistical Analyses

Statistical Package for Social Sciences 27.0 (SPSS) was used for all statistical analyses. Two-tailed analyses are used throughout. First, relations between analysis were examined with Pearson correlation. In order to understand child language development in more detail, regression models examine the relative contribution of the factors examined here. One regression model per language variable (dependent variable respectively: vocabulary, grammar, and pragmatics). A regression was chosen to fit the variables under consideration, with a stepwise selection of variables. From the pool or variables, the one that added most to the explained variance of the model was chosen first. Then the process iterated until all variables that contributed significantly to the explained variance were included in the model, while checking at each step if variables that do not contribute significantly to the model can be removed. All three regression models started with the same pool of variables.

## Results

The result section is organized according to our three main research questions. To address our first research question, we first report descriptive data of parent and child media use, JME, and the children’s language development scores for vocabulary, grammar, and pragmatics. Then to address our second and third research questions we describe a series of first-order correlations we conduced regarding the child’s and parent’s media use and the relation to the child’s language development, as well as the relation of language (i.e., vocabulary, grammar, and pragmatics) to interactional turn-taking, book reading, and JME. Finally, in order to understand vocabulary, grammar, and pragmatic development better, regression models examine the relative contribution of parent and child media use, interactional turn-taking, and JME to language development. One stepwise regression model per language variable is fitted.

### Research Question 1: Descriptive Statistics for Family Media Use, Home Environment, and Language

#### Estimated Daily Use of Media

Children aged 2years used DM daily (see Table 1), 86% watch TV, 64% use a smartphone, 52% used a tablet, and 16% used a computer and nearly 100% of the parents read to their child on a daily basis (27% <30min, 52% 30–60min, 14% 1–2h). The question regarding books did not differentiate between e-books and print books. The question regarding books did not differentiate between e-books and print books. No child played video games at this age. Child’s media use patterns were highly correlated with parent’s media use patterns for most types of media (all *p’s* > 0.01). The only media pattern that did not correlate between parent and child use was smartphone use. Children’s media use did not differ between boys and girls (all *p’s* > 0.05) nor was it associated with the amount of childcare the children attended (all *p’s* > 0.05). Consistent with other studies of children in this age range viewing TV content was the most frequent form of DM exposure (Rideout, 2017; Rideout and Robb, 2020). This was irrespective of the device used for viewing the TV content. Twenty-five percent watched TV on a digital device daily or several times a day, 19% of the children watched TV on a mobile device several times a week, and 56% one time a week or less (see Table 2). Daily use of e-books (8%) or playing games on mobile devices (5%) were not common.

**Table 1:** Children’s daily media use by type of device in percentage per time category (*n* = 88–92).

Media	No use	0–30min	30–60min	>60min
TV	14	22	36	28
PC	84	10	6	0
Tablet	48	26	19	8
Smartphone	36	45	17	2
Books^PandE^	1	28	56	16

**PandE:** This question refers to both print and e-books.

**Table 2:** Usage of digital devices for different digital media (DM) activities in percept per time category (*n* = 90).

Percentage of children who use digital device to watch	Do not use	Once a week	Several times a week	Daily – several times a day
TV	45	11	19	25
Movies	56	9	19	7
Play games	62	17	16	6
Video chat	49	24	23	3
Digital books	87	5	0	8
Music	44	19	23	15

#### Parental Use of Digital Media During Child Routines

Parents reported that they commonly used DM during child routines, such as mealtimes or bedtimes, but there are large individual differences (see Table 3). For subsequent analyses, values summarized into a mean of how likely the parent was to use DM during most child routines; would never use devices (7%), not very likely to use devices (51%), neither likely or unlikely to use devices (36%) and were likely to use devices (7%) during child routines. None of the parents selected the option “very likely” for all of the activities. Parents, furthermore, reported that they seldom had background TV on; 60% reported never or almost never, 24% reported sometimes, 13% reported often, and 3% reported always.

**Table 3:** Percentage of parents stating how likely it is that they would use a digital device during common everyday routines when their child is present (*n* = 90).

Activity	Never do this	Not very likely	Neutral	Likely	Very likely
During mealtime	26	54	11	7	2
Getting ready	32	51	7	11	0
During playtime	3	21	29	44	2
During bedtime	48	20	8	4	10
During transport	17	34	14	29	6

#### Media Mediation Strategies

For clarity, all three mediation strategies of the scale are presented but based on the review of the literature we hypothesized that only the JME strategy would be associated with language outcomes and therefore only this strategy is used in the correlational and regression analysis. Parents (*n* = 91) used different media mediation strategies when viewing TV content with their child. Restrictive mediation was the most common strategy among parents of 2-year-old’s (*M* = 3.22, *SD* = 0.89). That is, the parents decided when, how much and the content of the DM the child could use. The other two strategies were equally common, social co-viewing, viewing together without discussing the content (*M* = 2.78, *SD* = 0.71) and JME (*M* = 2.79, *SD* = 0.92).

#### Language Development (SECDI-2)

Language development of the 2-year-olds included in the present study (see Table 4) is consistent with Swedish norms of language development (Berglund and Eriksson, 2000). No difference was observed in any of the language measures depending on how many hours of childcare the children attended (all *p’s* > 0.05). Furthermore, no gender differences were observed on the grammar scale or for the vocabulary scale (*p’s* > 0.05). The girls had a significant higher mean than the boys on the pragmatic scale [girls: *M* = 8.84, *SD* = 1.9, boys: *M* = 7.4, *SD* = 1.6; *t*(86) = −2.57, *p* < 0.05]. All the language measures of SECDI-2 correlated with each other. The vocabulary scale correlated with the grammar scale (*r* = 0.77, *p* < 0.01) and with the pragmatic scale (*r* = 0.58, *p* < 0.01). The grammar scale correlated with the pragmatic scale (*r* = 0.59, *p* < 0.01).

**Table 4:** Descriptive data for three subscales of the Swedish Early Communicative Development Inventory – Words and Sentences (SECDI-2).

Variables	*M*	S*D*	Min-Max	Percentile score
Vocabulary scale (*n* = 90)	305.2	155.9	8–586	54
Pragmatic scale (*n* = 88)	7.9	1.8	2–10	50
Grammar scale (*n* = 89)	5.6	3.2	0–12	55

#### Home Sound Environment (LENA)

Language ENvironment Analysis records the sound environment of the child and then uses tested algorithms to break the audio record into different categories. These categories include a number of words spoken to the child by the adult and the number of child vocalizations. Then based on the child and adult words and the time between the utterances, turn-taking between the two is calculated. Variation in how many words spoken by adults and the number of interactional turns was large, from about 4,000 to 34,000 words a day with a mean of 17,267 words and a mean of 829 episodes of turn-taking during a day (see Table 5). These variables are mutually dependent and adult word count was positively correlated with child word count (*r* = 0.28, *p* < 0.01) and interactional turn-taking (*r* = 0.68, *p* < 0.01). Interactional turn-taking correlated with child word count (*r* = 0.81, *p* < 0.01). In the subsequent analysis interactional turn-taking will be utilized as it contains both adult word count and child word count and the measures are highly intercorrelated, but for clarity descriptive statistics for both those measures were reported here.

**Table 5:** Descriptive data for three measures of the home sound environment obtained from the Language ENvironment Analysis (LENA, *n* = 84).

Variables	*M*	*SD*	Min-Max
Adult word count	17,267	6,898	4,228–34,418
Child word count	3,178	1,422	390–7,582
Turn-taking	829	387	100–1,721

### Research Questions 2 and 3: Can Language Development Be Predicted by Family Media Usage Patterns and Factors in the Home Environment Such as Adult-Child Turn-Taking, Book Reading (e-Books or Paper Books), and JME?

#### Correlational Analysis

In line with our second question, there was a significant negative correlation between how much the child watched TV content (defined as any TV content viewed on any device from any source) and the vocabulary scale (see Table 6). There was no correlation between the child’s use of smartphones and tablets, and the child’s language development (*p’s* > 0.05). Computers were used by 14 of the children, but there was a significant positive correlation between the child’s use of computers and vocabulary.

**Table 6:** Correlations between three measures of language development, and child media use, parental DM use, parental mediation and home sound environment (LENA).

Vocabulary		Grammar	Pragmatic
**Child media use (*n* = 89–90)**			
TV content	−0.27**	−0.08	0.05
Tablet	0.01	0.06	−0.04
Smartphone	−0.09	0.01	0.12
Computer	0.21*	0.08	0.01
Books^PandE^	0.21*	0.20	0.28*
**Parental media activities (*n* = 89)**			
Likelihood of DM use	−0.27*	−0.22*	−0.30*
Background TV	−0.16	−0.01	−0.04
JME	0.15	0.18	0.29**
**Home sound environment (*n* = 78)**			
Turn-taking	0.41**	0.18	0.20

* *p < 0.05*.

** *p < 0.01*.

**PandE:** This question refers to both print and e-books.

Furthermore, a correlation analysis between the parent’s DM use (smartphones, computers, tablets TV, and videogames) and the child’s language development revealed no significant associations (all *p’s* > 0.05). This line of questions pertained to the parent’s use of DM when the child was present but also when the child was not present. There was, however, a significant negative correlation between higher likelihood of parental use of DM during child routines and the child’s vocabulary, grammar, and pragmatic scale (see Table 6). Background TV was not common in our sample and did not correlate with language measures (*p’s* > 0.05).

Our third research question addressed whether parent-child activities at home were related to language. JME was positively correlated with the child’s developing pragmatics ability (see Table 6). There was also a positive correlation between the children being read to and both the pragmatic and vocabulary scale. In our original research question, we equated print books and e-book. E-books were, however, not commonly used in this sample therefore it is likely that this correlation is largely due to reading print books. Furthermore, adult-child interactional turn-taking, as measured with LENA, was positively correlated with the child’s vocabulary.

#### Regression Models

We built three separate stepwise regression models to predict the language variables: Vocabulary scale, pragmatic scale, and grammar scale, respectively. For further analysis see multiple linear regressions in the appendix. Predictor variables were the same in all three regressions and were chosen based on the first order correlational analysis and whether they were theoretically likely to predict language outcomes. From the CAFE questionnaire, we consequently included the time the child spent with books, TV content, and computers and for these, dummy variables were created with a median value as the reference category, based on the incidence of use of the specific media. TV content was recalculated into three variables of daily use: no TV, low TV (<60min), and high TV (>60min). Computer use was recalculated into no use, medium use (<30min), and high use (30–60min), Book reading was also recategorized into three variables: no/low use (<30min), medium use (30–60min), and high use (>60min). Medium TV, medium computer, and medium book use were used as the reference category, respectively. We also included the likelihood of parent’s device use during daily child routines, as well as the parent’s use of JME during child’s DM use. From the sound environment at home, measured with LENA, we included interactional turn-taking as a predictor variable. We also included gender as a predictor variable. As parental education did not have a normal distribution (over 80% of the parents had a university degree), this variable was not entered into the analysis. Additionally, as only 20% of the responders were fathers, therefore, the gender of the parent responding to the questionnaire was not entered into the model.

##### Regression 1. Vocabulary Scale

We entered the predictor variables into a linear stepwise regression on the vocabulary scale from the SECDI-2 as the outcome variable. The final model, based on three variables (see Table 7) was clearly significant *F*(3,69) = 9.87, *p* < 0.001, and explained a relatively large portion of the variance (adjusted *R^2^* explaining 27% of the variance). The model included three variables; interactional turn-taking which explained 16% of the variance, TV content, which explained 9% of the variance and the likelihood of parent’s device use during daily child routines which explained 5% of the variance.

**Table 7:** Summary of stepwise regression analysis for variables predicting SEDCI-2 – Vocabulary (*n* = 76).

	Step 1			Step 2			Step 3		
	B	SE B	*β*	B	SE B	*β*	B	SE B	*β*
Turn-taking (LENA)	0.15	0.04	0.40**	0.12	0.04	0.32**	0.10	0.04	0.28*
TV content No TV				130.26	45.77	0.31**	138.35	44.67	0.33**
Device use							−40.33	18.08	−0.23*
*R^2^*	0.16			0.25			0.30		
*F* for Δ*R^2^*	13.83**			8.1**			4.98*		
*ΔR^2^*	0.16			0.09			0.05		

* *p < 0.05*.

** *p < 0.01*.

**Table 8:** Summary of stepwise regression analysis for variables predicting SEDCI-2 – Grammar (*n* = 76).

	Step 1			Step 2		
	B	SE B	*β*	B	SE B	*β*
TV content No TV	2.87	1.01	0.32**	2.94	0.98	0.33**
Device use				−0.90	0.41	−0.24*
*R^2^*	0.10			0.16		
*F* for Δ*R^2^*	8.08**			4.8*		
Δ*R^2^*	0.10			0.06		

* *p < 0.05*.

** *p < 0.01*.

**Table 9:** Summary of hierarchical regression analysis for variables predicting SEDCI-2 – Pragmatics (*n* = 76).

	Step 1			Step 2			Step 3		
	B	SE B	*β*	B	SE B	*β*	B	SE B	*β*
Device use	−0.62	0.22	−0.31**	−0.6	0.22	−0.30**	−0.48	0.22	−0.24*
Gender				1.0	0.37	0.29**	1.1	0.37	0.31**
JME							0.43	0.20	0.23*
*R^2^*	0.10			0.18			0.23		
*F* for Δ*R^2^*	7.8**			7.2**			4.4*		
Δ*R^2^*	0.10			0.08			0.05		

* *p < 0.05*.

** *p < 0.01*.

##### Regression 2. Grammar Scale

Once again, we entered the same predictors into a linear stepwise regression with a grammar scale from the SECDI-2 as the outcome variable (see Table 8). The overall model which included two variables was significant [*F*(2,70) = 6.68, *p* < 0.01] with an adjusted *R^2^* of 14%. TV content explaining 10% of the variance and the likelihood of parent’s device use during daily child routines explained 6%.

##### Regression 3. Pragmatic Scale

Finally, the same predictors were added into a linear stepwise regression with the pragmatics scale from the SECDI-2 as the outcome variable (see Table 9). The final best fit model was significant [*F*(3,69) = 6.92, *p* < 0.001] with an adjusted *R^2^* explaining 20% of the variance in the pragmatic scale and included three variables. The likelihood of parent’s device use during daily child routines explained 10% of the variance, gender of the child explained 8% of the variance and JME explained an additional 5% of the variance.

## Discussion

The present study confirms that specific aspects of the child’s media environment were associated with the child’s language development. The time spent in interaction with an adult was positively associated with the development of language. The parent’s tendency to use DM during childhood routines, as well as the time children spent watching TV content was negatively associated with the child’s language development.

### What Is the Media Environment and Language Environment of 2-Year-Old Swedish Children?

Consistent with other recent reports (e.g., Rideout, 2017), toddlers in Sweden watch TV shows on a number of devices, on a TV or tablet/cellphone, and view content from multiple sources. Consistent with other recent reports of data collected in the United States (e.g., Rideout, 2017; Rideout and Robb, 2020) and Canada (e.g., Madigan et al., 2020), media use in Swedish 2-year-old was frequent. Children in the present study actively consumed different forms of media daily, utilizing a number of different devices: TV, smartphones, tablets, as well as books. Playing games or video chat, however, was not that common. The frequency of parent-child interactions varied between families and our findings suggest that parents are the originators of the child’s home language environment and media ecology (Nathanson, 2015; Nansen and Jayemanne, 2016; Barr, 2019). Some parents were very talkative, whereas others did not interact that much, and the number of adult words directed toward the child varied from as little as 4,000 words to as many as 34,000 words a day. Children participated in interactional turn-taking, on average, 829 times a day with an adult. Once again, there were large individual differences with rates of turn-taking varying from as low as 100 to almost 2,000 interactional turns a day. The amount of language input that the children experienced, thus, varied immensely. Nevertheless, the language development of children in this study was within the typical norms.

The media habits of the parents are mimicked in the emerging media habits of the child. The time parents spend watching TV or reading books was correlated with the time the children spent watching TV or being read to, respectively. Children were read to daily, but e-books were not common. The parents reported that they frequently used DM in routine situations with the child. They also reported that they try to mediate their child’s media use. The most common mediation strategy that parents reported at this age was to restrict DM use. But co-viewing or JME were common strategies as well.

### Are 2-Year-Old’s DM Use and Parents’ DM Use Associated With the Child’s Language Development?

Our second research question and accompanying hypotheses were partially confirmed. The child’s DM use was associated with the child’s language development. The parent’s overall DM use was not correlated with the child’s language development, but the likelihood that parents use DM in child routines was associated with the child’s language development.

An increase in the child’s use of DM was negatively associated with language development, specifically the vocabulary and grammar scales. The regression showed that significant variance of the children’s grammar development (10%) and vocabulary development (9%) can be uniquely predicted by the time the child spent watching TV content. Although the children in this study watch a fairly moderate amount of TV content, 58% of the 2-year-olds watch TV content 1h or less a day – such exposure was associated with variation in language development. Earlier studies have shown that increased TV watching was associated with a decrease in language proficiency for children up to 2years of age (Zimmerman et al., 2007; Madigan et al., 2020). The current study shows that watching TV may interfere with language learning after 2years of age as well. It is important to note that the results do not point to an atypical delay in language development in 2-year olds. As a group, the children perform within the norms of typical development (Berglund and Eriksson, 2000), but one explanation for the variation in language development within this sample is their consumption of TV content on a TV, tablet, or smartphone.

It is also worth noting that we cannot make a strict distinction as to whether the children watch TV on a big screen, a tablet, or a smartphone screen. It was very common in our sample to watch TV on handheld screens and young children, view TV content on several different devices. At this age, games and educational apps are not as common as watching TV content. Unfortunately, the lack of information on the specific content of media exposure is a limitation of the current study. Additional studies should more closely examine the TV content that toddlers are exposed to in order to provide a finer-grained analysis of the types of content that are negatively associated with child language development. There was also a correlation between computer use and vocabulary, although not significant in the stepwise regression. It is difficult to know the reason for this association due to the small number of children who use computers and since the regression did not show any significance it is not a robust finding. Knowing the content of the computer use could have explained this association. One could speculate, for instance, that if the children had used the computer to video chat this could explain the positive correlation to language. About half the sample uses video chat at least once a week. The current COVID-19 pandemic has once again changed patterns of interaction and reports suggest that video chat rates are much more frequent than before the pandemic (Gaudreau et al., 2020). Future studies should examine how these increased levels of video chat are associated with language outcomes and whether video chat is differentially associated with changes in pragmatics or vocabulary or if there are gender differences.

We did not find an association between the parent’s overall use of digital devices and the child’s language development. Rather, it was specifically the likelihood of parental use of DM use during child routines which was negatively associated with all three measured aspects of child language development, vocabulary, grammar, and pragmatics, in line with our hypothesis. The stepwise regression analysis demonstrated that the likelihood of parental DM use during child routines predicted variation in vocabulary (5%), grammar (6%), and in pragmatic ability (10%). Parental use of DM during child routines may lead to technoference in interactions with the child, as previous research has shown (McDaniel and Radesky, 2018a). In situations, such as mealtime, getting ready, or bedtime about 80% of all parents reported that they did not use DM, whereas 20% reported using DM during these routines. In other situations, such as during transport and during playtime the numbers who reported using their DM device was considerably higher. During playtime, for instance, 75% of parents reported using their own smartphone at least some of the time. This is consistent with findings showing associations with technoference and other child outcomes, such as self-regulatory behaviors and external behavior problems (McDaniel and Radesky, 2018a; Sundqvist et al., 2020). Parental report of DM use during child routines was not a measure of technoference, *per se*, as we did not ask them if they had experienced technoference in these situations. However, as it may be difficult to always remember and accurately analyze all the cases of technoference experienced in a day, simply asking about and pinpointing certain situations of DM use might be a feasible proxy of rate of technoference. Future studies could more closely examine parental media use using passive sensing technology to more accurately measure bursts of parental DM usage (Barr et al., 2020; Milkovich and Madigan, 2020; Radesky et al., 2020). The parent’s background TV is another DM use that has been seen to be a negative factor for the child’s attention and learning (Pempek et al., 2014). The parents in this study did, however, not use background TV to a great extent. This is an interesting line of further study as it may indicate that not only digital sounds can be distracting but parent’s “silent use” of DM and withdrawal of their attention may also interfere with language development.

Parents are the creators of the child’s media and language learning environment (Nathanson, 2015; Nansen and Jayemanne, 2016; Barr, 2019). The actions of the parents, rather than other factors, seem to matter the most. All the children in this study attended state subsisted childcare daily, with most children attending more than 20h a week. Unlike other studies (Shivers and Barr, 2007), childcare exposure was, thus, held fairly constant across participants in this study and was not found to be a relevant factor. Time spent in childcare outside the home was not associated with either the children’s DM use or language development. There might, of course, be other differences in the different caregivers that will affect the child’s development even though the number of hours the children attended care outside of the home did not. Although the parent’s own overall media use did not correlate with the child’s language development, it is evident that the child’s language learning, at this age, is closely associated with parents’ actions and the home environment. Future studies should examine the media environment of the childcare language and media environment to examine how home and childcare factors overlap.

### Are Parent-Child Activities in the Home Environment Associated With the Child’s Language Development?

Our third study question and associated hypotheses were partially confirmed. Parents’ use of JME was positively associated with language development, specifically pragmatic abilities. Talking and interacting with your child when viewing DM seems to be important and uniquely explained 5% of the variation in the understanding of pragmatics. Consistent with Beyens et al. (2019), this signified the importance of not just co-viewing media with your child but also interacting around the media content as well. Talking about the intent of the actors and explaining their actions and feelings are aspects of JME and could be considered specific linguistic input that most certainly would be related to pragmatic understanding. These findings suggest that JME may be an effective strategy for maintaining language input during media exposure. Additional longitudinal research more closely examining the content and context of media exposure is warranted to examine longer-term implications of JME.

We measured the child’s sound environment at home with the digital recorder LENA analyzing conversational turns between the child and the adult. Conversational turn-taking between child-adult is important for language development (Romeo et al., 2018), and for the children in the present study, it explains 16% of the variance in vocabulary, thus, confirming the important role of child-adult interactions. As previous studies have shown, one of the most important aspects of the interactions between the child and the adult is that the interactions are immediate, reliable, and accurate in content and relevance (Roseberry et al., 2014). This attentive communication style maintains an environment that is stimulating for language development (Karrass and Braungart-Rieker, 2003) and an increase in the interactions between the child and the parent is associated with an increase in the child’s language development. That is, engaging in more parent-child interactions was associated with faster vocabulary growth.

Consistent with prior research, the current study shows that media use, as well as the interactive linguistic patterns, seems to “run” in families (Nansen and Jayemanne, 2016). The child’s use of DM and books were correlated with the parents’ own use of the same media. Parents who themselves read more are also prone to read more to their children (Nathanson, 2015). Almost all children in this study were read to on a daily basis, and as previous studies have shown, this is associated with their parent’s own literacy habits (Karrass et al., 2003; Chaparro-Moreno et al., 2017). E-books were not commonly read by families in this study. Adults and children alike mostly read paper books.

The fact that this well-educated group of parents reads to their children on a daily basis might explain the fact that time spent in book reading did not account for unique variation in language in the regression analysis although the book reading was correlated with pragmatics abilities. Thus, partly confirming our hypothesis that reading is associated with language development and possibly explaining the lack of significance in the regression. This finding suggests that unique variance may also be due to likely differences in the interactional quality during book reading and other activities. Future studies of the language environment can take advantage of transcripts from the LENA recordings to provide a more in-depth analysis of interactional quality during different everyday events involving both DM and book reading.

Analyzing the different aspects of language measured here Vocabulary (Content), Grammar (Form), and Pragmatics (Use) it is evident that they have several commonalities as well as factors that differ between them. The developmental trajectory of pragmatics is earlier than vocabulary and grammar, but the three aspects are intercorrelated, yet different aspects of the environment are associated with different language outcomes.

The child’s TV content watching seems to be negatively associated with the development of vocabulary and grammar. The parent’s device use during child routines seems to be negatively associated with the development of vocabulary, grammar, and pragmatics. The adult-child interactions were positively associated with the development of vocabulary. The development of pragmatics seemed to be dependent on several different factors including the gender of the child, parents’ device use during child routines, and JME. It is important to note that our results say nothing about causality. It may be that parents talk less to the child just because the child’s language skills are less developed, or that a parent will use their cell phones more when they are with their child since the child does not speak as much. There might also be other stressors in the family, for instance, child behavior or parent’s work situation that contribute to the media usage and the child’s language development (e.g., McDaniel and Radesky, 2018a; Sundqvist et al., 2020).

None of the measures of media use differed significantly between boys and girls. Boys and girls were reported to use as much DM, their parents used as much DM and there are no differences in terms of how parents mediated use between boys and girls. Girls did, however, show more advanced pragmatic ability compared to boys. Previous studies (Westerlund and Lagerberg, 2008) have also shown that girls in this age range may develop aspects of language faster than boys, but this difference does not seem to be mediated by media use.

A limitation of the present study is the construction of some of the media questions, which made a distinction between watching TV and other devices such as smartphones and tablets but not between the content being watched. Between the time when the questions were constructed and the time when they were asked it became increasingly common to watch TV on a tablet or stream from a tablet to the TV. This question will no longer easily distinguish between handheld DM and stationery viewing devices nor between the content used on different devices, for instance, if the computer is used for video chat or TV content. Future studies should instead focus on disambiguating the content and context of media usage rather than focusing on the device that is being used. Furthermore, the questions regarding time spent using different content and different devices were difficult to use and should have been on a continuous scale for more optimal analysis. One possibility would be to use a passive sensing app on the devices the child uses along with a parental report diary (Barr et al., 2020). Finally, it is also important to note that this sample consisted of parents of relatively high SES and the results may not be generalizable to a group of parents with lower SES.

Our study confirms that specific aspects of the child’s DM environment are associated with child language development. Children at age 2years actively consume different forms of media daily *via* TV, smartphones, tablets, as well as books. More TV content, whether it is viewed on a big screen or tablet is negatively associated with language development. The likelihood of parent’s device use during child routines was also negatively associated with the child’s language development. Positive linguistic parental strategies such as interactional turn-taking with the child, book reading, and JME when watching DM, on the other hand, were positively associated with the child’s language development.

## Data Availability Statement

The raw data supporting the conclusions of this article will be made available by the authors, without undue reservation.

## Ethics Statement

The studies involving human participants were reviewed and approved by the Research Ethics Review Committee at Linköping University. Written informed consent to participate in this study was provided by the participants’ legal guardian/next of kin.

## Author Contributions

The study was conceived by AS, F-SK, RB, and MH. AS, F-SK, and UT coordinated the acquisition of data and collected data. AS analyzed the data and drafted the manuscript. All authors contributed to the article and approved the submitted version.

### Conflict of Interest

The authors declare that the research was conducted in the absence of any commercial or financial relationships that could be construed as a potential conflict of interest.

The reviewer ADR and handling editor declared their shared affiliation. The reviewer ADR declared a past collaboration with the authors to the handling editor.
