# Supplementary material for: Growing Up in a Digital World – Digital Media and the Association With the Child’s Language Development at Two Years of Age
Source: Front Psychol. 2021 Mar 18;12:569920. doi: 10.3389/fpsyg.2021.569920 (PMC8015860; doi:10.3389/fpsyg.2021.569920)
Supplement: Supplementary file 1 [file Table_1.docx]

To confirm the results from the stepwise regressions we built three separate linear regression models to predict the language variables: Vocabulary scale, Pragmatic scale, and Grammar scale, respectively. Predictor variables were the same as in the stepwise regressions and the same in all three linear regression; the time the child spent with books, TV content, and computers and for these, dummy variables were created with a median value as the reference category, based on the incidence of use of the specific media. TV content was recalculated into three variables of daily use: no TV, low TV (< 60 min), and high TV (> 60 min). Computer use was recalculated into no use, medium use (< 30 min), and high use (30-60 min), Book reading was also recategorized into three variables: no/low use (< 30 min), medium use (30-60 min), and high use (> 60 min). We also included the likelihood of parent’s device use during daily child routines, as well as the parent’s use of JME during the child’s DM use. From the sound environment at home, measured with LENA, we included interactional turn-taking as a predictor variable. We also included gender as a predictor variable. The analysis with linear regressions confirms the overall pattern of predictor variables associated with language measures, these predictor variables are TV content, interactional turn-taking, likelihood of parent’s device use during daily child routines, JME, and gender.

A significant regression equation was found with the vocabulary scale, *F*(10,62) = 4.1, *p* < .001, and explaining a relatively large portion of the variance (adjusted *R2* explaining 40% of the variance). The variables that were significant were; interactional turn-taking (*B* = 0.9 *p* < .05), TV content (*B* = 158, *p* < .01), and the likelihood of parent’s device use during daily child routines (*B* = -46.6, *p* < .05). The remaining variables were not significant : Gender (*B* = 41.1, n.s.), JME (*B* = 27.2, n.s.), books (low/high) (*B* = -41.34/-53.41 n.s.), PC (no/high) (*B* = -17.48/36.56, n.s. TV high (*B* = 33.64, n.s.).

A significant regression equation was found with the pragmatic scale, *F*(10,62) = 2.35, *p* < .05, with an  *R2* explaining 27% of the variance). The variables that were significant were: Gender (*B* = 1.15, *p* < .01 and JME (*B* = .47, *p* < .05). The remaining variables were not significant: books (low/high) (*B* = -.3/.27, n.s.), PC (no/high)(*B* = -.58/

-.10, n.s. TV high (*B* = .40, n.s.) interactional turn-taking (*B* = .01, n.s,), likelihood of parent’s device use during daily child routines (*B* = -.39, n.s.), TV content no/high (*B* = 0.94/.40, n.s).

A significant regression equation was not found with the grammar scale, *F*(10,62) = 1.74, *p* = .09. The variables that were significant were; TV content no/high (*B* = 3-41/1.8 *p* < .05. The remaining variables were not significant Gender (*B* = .06, n.s,) JME (*B* = .24, n.s), Gender (*B* = .06 ,ns), books (low/high) (*B* = -.64/-.42, n.s.), PC (no/high)(*B* = .10/-01, n.s, interactional turn-taking (*B* = .001, n.s,), likelihood of parent’s device use during daily child routines (*B* = -.82, n.s.), TV content no/high (*B* = 3.41/1.8 n.s).

*Summary of* Multiple Linear *Regression Analysis for Variables Predicting SEDCI-2 – Vocabulary, Pragmatics and Grammar (n=76*)

|  | Vocabulary | | | Pragmatics | | | Grammar | | |
| --- | --- | --- | --- | --- | --- | --- | --- | --- | --- |
|  | *B* | *SE B* | *β* | *B* | *SE B* | *β* | *B* | *SE B* | *β* |
| Turn-taking | .09 | .02 | .23* | .00 | .00 | .07 | .00 | .00 | .07 |
| TV content |  |  |  |  |  |  |  |  |  |
| No TV | 158 | 46.72 | .37** | .94 | .58 | .2 | 3.41 | 1.12 | .38** |
| High TV | 33.64 | 35.84 | .1 | .40 | .44 | .11 | 1.8 | .86 | .25* |
| Device use | -46.6 | 22.2 | -.22* | -.39 | .28 | -16 | -.82 | .53 | -.18 |
| JME | 27.2 | 17.68 | .16 | .47 | .22 | .25* | .24 | .43 | .07 |
| Gender | 41.1 | 33.48 | .13 | 1.15 | .42 | .33** | .06 | .81 | .01 |
| Book use |  |  |  |  |  |  |  |  |  |
| Low | -41.34 | 36.53 | -.12 | -.30 | .45 | -.80 | -.64 | .88 | -.91 |
| High | -53.41 | 44.93 | -.13 | .27 | .56 | .06 | -.42 | 1.08 | .38 |
| Computer |  |  |  |  |  |  |  |  |  |
| No | 36.56 | 75.54 | .06 | -.09 | .93 | -.01 | .10 | 1.82 | .008 |
| High | -17.49 | 53.26 | -.04 | .58 | .66 | .13 | -.01 | 1.28 | -.01 |
|  |  |  |  |  |  |  |  |  |  |
| Adjusted *R^2^* |  | .30 |  |  | .16 |  |  | .09 |  |
| F for Δ*R^2^* |  | 4.1** |  |  | 2.35* |  |  | 1.74 |  |
| Δ*R^2^* |  | .40 |  |  | .27 |  |  | .22 |  |

**p* < .05. ***p* < .01
